# Cancer Stem Cell-Associated Immune Microenvironment in Recurrent Glioblastomas

**DOI:** 10.3390/cells11132054

**Published:** 2022-06-28

**Authors:** Yoshitaka Murota, Kouichi Tabu, Tetsuya Taga

**Affiliations:** Department of Stem Cell Regulation, Medical Research Institute, Tokyo Medical and Dental University (TMDU), 1-5-45 Yushima Bunkyo-ku, Tokyo 113-8510, Japan; muro.scr@mri.tmd.ac.jp

**Keywords:** glioblastoma, recurrence, cancer stem cell, immune microenvironment

## Abstract

Glioblastoma multiforme (GBM) is the most incurable tumor (due to the difficulty in complete surgical resection and the resistance to conventional chemo/radiotherapies) that displays a high relapse frequency. Cancer stem cells (CSCs) have been considered as a promising target responsible for therapy resistance and cancer recurrence. CSCs are known to organize a self-advantageous microenvironment (niche) for their maintenance and expansion. Therefore, understanding how the microenvironment is reconstructed by the remaining CSCs after conventional treatments and how it eventually causes recurrence should be essential to inhibit cancer recurrence. However, the number of studies focusing on recurrence is limited, particularly those related to tumor immune microenvironment, while numerous data have been obtained from primary resected samples. Here, we summarize recent investigations on the immune microenvironment from the viewpoint of recurrent GBM (rGBM). Based on the recurrence-associated immune cell composition reported so far, we will discuss how CSCs manipulate host immunity and create the special microenvironment for themselves to regrow. An integrated understanding of the interactions between CSCs and host immune cells at the recurrent phase will lead us to develop innovative therapies and diagnoses to achieve GBM eradication.

## 1. Introduction

Glioblastoma multiforme (GBM) is the most lethal adult primary brain cancer and remains incurable despite intensive treatments. The standard treatment of newly diagnosed GBMs involves chemo/radiotherapies following maximum safe surgical resection. Unfortunately, the highly invasive nature of GBMs prevents the complete removal of cancer cells and leads most patients to relapse eventually [1]. The invasive GBMs display complex cellular and molecular networks by recruiting various immune cell subsets that play critical roles in tumor recurrence [2]. However, as most experimental and translational studies have been based on primary resectable tumors, understanding the specific properties of recurrent tumors and their microenvironment will provide us innovative strategies to tackle resistance to conventional treatments and regrowth of GBMs. 

Some have referred to the potential mechanisms of GBM recurrence mainly by focusing on the genomic and molecular landscape [3,4]. Of particular interest is the study by Kim et al., that proposed two recurrence models by comparing the genomic profiles of rGBMs and their original primary specimens. The first is the “clonal evolution model” describing recurrence by primary clones that acquired a competitive advantage for surviving treatments and regrowing eventually. In this model, all mutations in the surviving primary clones are preserved in the recurrent specimens, and additional mutations are newly accumulated and detected in the recurrent tumor [5]. In this case, the recurrent tumors are likely to develop from the therapy-resistant clones at the primary phase and, therefore, mutations that confer high survivability and adaptivity are inherited and subsequently expanded at recurrence. For example, a comparison of the genomic landscape of 23 patients with low-grade gliomas at initial diagnosis and patient-matched recurrence demonstrates that 54% of mutations in the initial tumors are undetected in the recurrent ones, and that the adjuvant chemotherapy with the anti-glioma drug temozolomide (TMZ) is mutagenic in some cases to confer new driver mutations in the RB and AKT-mTOR pathways [6,7]. A type of recurrence not applicable to the clonal evolution model is explained by the “ancestral cell origin (cancer stem cell) model”, which describes the presence of refractory ancestral cells. After therapeutic removal of even all the daughter cells, the remaining ancestor accumulates new mutations and proliferates to become the recurrent tumor. In this model, mutations shared by primary and recurrent tumors are therefore presumably only from the ancestral cell, and the subsequent primary and recurrent tumors are thus more divergent [5]. Since the tumor is composed of cells that are heterogeneous, recurrence may not be exclusively based on either of these two models, but it may occur in a concoction of the two.

Given the poor execution of the current molecular-targeted therapies against rGBM, an integrated understanding of the interplays between GBM cells and non-neoplastic cells in this malignant disease is imperative [8]. Here, we review the literature on immune cell components and how the microenvironments that support cancer regrowth are configured in rGBM. We also discuss cancer stem cell (CSC) behaviors in the context of recurrence, especially how they evade host immune attacks and create a self-advantageous microenvironment. 

## 2. Conventional Approach to Elucidate Immune Microenvironment in rGBMs

The microenvironment of GBM tumors includes not merely resident components of the brain such as neurons, glial cells, and microglia but also brain-infiltrating myeloid-derived cells. These components contribute to GBM progression by releasing soluble factors associated with the growth promotion of cancerous cells, ECM remodeling, and angiogenesis, etc. In addition, the anti-tumor immune compartment is exhausted in the microenvironment with the consequent feature of immunosuppression [2]. Lately, studies that uncover the immune microenvironment in rGBMs are slowly coming into our view, and it looks unique when compared with that observed in the primary ones. 

To investigate the immune microenvironment of rGBMs, Antunes et al., performed a single-cell RNA sequence (scRNAseq) analysis of CD45+ immune cells in fresh tumor specimens obtained from seven patients with newly diagnosed (ND) and four patients with recurrent diseases [9]. While all patients with ND disease were treatment-naïve, patients with disease recurrence had previously undergone surgical resection and conventional chemo/radiotherapies. They found more diverse immune compartments in the rGBM microenvironment, which include the increased T cells, natural killer (NK) cells, B cells, and monocytes. Importantly, they reported that tumor-associated macrophages (TAMs) with microglial gene signatures were predominant in ND tumors but replaced by ones with monocyte gene signatures following recurrence. The comparison between TAMs before and after conventional treatments revealed several genes related to monocyte chemotaxis, interferon (IFN)-signaling, and phagocytosis at the recurrent phase [9]. The exposure to interferon-γ is known to epigenetically change the gene expression profiles of GBM cells to immune-evasive phenotypes [10], and the formation of “TAM-GBM hybrids via phagocytosis renders GBM cells more invasive [11,12,13] (as discussed in Section 5), strongly suggesting that monocyte-derived TAMs (Mo-TAMs) are associated with recurrence. 

Similar to work by Antunes et al., Fu et al., took advantage of mass cytometry by time-of-flight spectrometry (CyTOF) to conduct multi-parameter single-cell protein analysis of CD45+ immune cells localized in tissues from three rGBM patients [14]. The immunosuppressive subgroup characterized by the high expression of indoleamine 2,3-dioxygenase 1 (IDO1; [15]) and low expression of MHC class II molecule (HLA-DR) was identified in rGBMs as one sort of mononuclear phagocytes, glioma-associated microglia, and macrophages (GAMs) [14]. This study did not identify whether this immunosuppressive GAM subgroup originated from resident microglia or infiltrating monocytes, but proposed that the proportion of the whole GAM population in CD45+ cells in rGBM is decreased compared with those in primary GBM (pGBM) and both share the immunosuppressive features. 

As the studies above suggest, GAMs (or TAMs) with immunosuppressive properties have an important role in recurrence, but the results of clinical trials were unsatisfactory. For example, PLX3397, a small molecule that selectively inhibits CSF1R associated with TAM development, in the Phase II study of patients with rGBM readily entered rGBM tumor tissues and decreased Iba1+ macrophages concomitantly with the elevated CSF1 ligand in peripheral blood; however, there was no significant improvement in progression-free survival (PFS) [16]. This outcome implied that monotherapy targeting macrophages is not fully sufficient to extend patients’ PFS in GBMs, and the development of new therapies targeting multiple immune cells including macrophages is imperative to achieve GBM eradication.

On the other hand, Mohme et al., focused on effector and memory T cell differentiation by performing flow cytometric analysis on paired samples of pGBMs and rGBMs [17]. The tumor-infiltrating lymphocyte (TIL) compartment in rGBMs displayed a significantly enlarged proportion of CD8+ and CD4+ effector memory T cells (T_EM_, defined by CD45RA-, CCR7-, CD28-) than those in pGBMs. Consistently, CD8+ TILs with the transitional memory T cell (T_TM_, defined by CD45RA-, CCR7-, CD28+) phenotype were less common in rGBM than in pGBM. They speculated these situations as a serially ongoing response to the recurrent tumor, since T_TM_ is a less differentiated type than the T_EM_ antigen-experienced population [18,19]. Noteworthy, their clustering analysis showed that the most strikingly different characteristics of TILs in rGBM were the high expression of PD-1 and other markers for T cell exhaustion, while PD-1 was also highly expressed in the peripheral blood lymphocytes (PBLs) in rGBM than pGBM. Although the proportion of T cells infiltrated into rGBM tumors is relatively high, they seem to be functionally exhausted. They further performed T cell antigen receptor (TCR) sequencing to compare antigen specificity and found the reduced diversity of TCR in the recurrent situation for both TIL and PBL compartments. When the CDR3 (complementarity determining region 3) amino acid sequence was compared between pGBM and rGBM, a trend toward the reduced diversity of the T cell repertoire was more prominent in rGBM than in pGBM. The proportion of initially identified TILs at the primary phase was comparatively low in rGBM samples. These data fully indicated that the composition of the immune repertoire is dynamically altered along with GBM progression [17]. T cell immune response requires antigen presentation that is dependent on MHC proteins. As reviewed by Brown et al., the MHC proteins are downregulated in glioblastoma, which results in immune escape [20]. Interestingly, the GAM subgroup observed by Fu et al., displayed less expression of HLA-DR. Further investigation of how the activities of antigen presentation are attenuated in GBM is needed. In fact, a Phase III clinical study demonstrated that anti-PD-1 monoclonal antibody Nivolumab was not superior to anti-VEGF monoclonal antibody Bevacizumab from the view of OS [21,22]. Additionally, anti-PD-1 therapy combined with surgical treatment effectively activates local and systemic immune responses to rGBM, but it is still far from eradication [23,24]. These data suggest that both immune checkpoint blockade and antigen presentation augmentation need to be exploited. In addition to activating T cells by anti-PD-1, we need a new perspective that improves antigen presentation, potentially leading to the improvement of TCR activation. Given the report that T cells are accumulated in the bone marrow rather than recruited to the GBM tissue due to the lack of Sphingosine 1-phosphate receptor 1 (S1P1) expressed on the surface [25], it seems meaningful to call up anti-tumor T cells onto tumor tissue before therapies targeting immune checkpoint molecules. 

Petterson et al., compared the gene expression between paired pGBMs and rGBMs based on the RNA obtained from formalin-fixed paraffin-embedded (FFPE) tissue sections [26]. Gene set enrichment analysis suggested the expression of genes involved in metabolism, extracellular matrix (ECM) remodeling, and complement activation are lower in rGBMs than pGBMs. Instead, genes related to T cell activation and checkpoint signaling were high in rGBM, suggesting T cell activation is part of the event that promotes the recurrence of GBM progression (we will discuss how this affects GBM in a later part). Moreover, they compared early recurring tumors (shorter than 14 months after primary treatments) with late recurring tumors (longer than 14 months after primary treatments) and found that genes involved in ECM remodeling, angiogenesis, and growth factor signaling are expressed higher, and genes related to Th2 cell activation, chemokine signaling, and toll-like receptor (TLR) are lower in tumors with early recurrence [26]. The differentially expressed genes between early- and late-recurrent tumors must provide useful information for predicting the patient outcome. 

## 3. Alternative Approaches to Elucidate the Immune Microenvironment in rGBM

### 3.1. Computational Analysis Using Large-Scale Omics Data of Recurrent GBMs

Even though the studies mentioned above strongly indicated that the immune cells are key components in GBM recurrence, the number of specimens obtained from rGBM patients is still quite small. Besides the limited number of rGBM samples, practical immunophenotyping methods such as immunohistochemistry and flow cytometric analyses also face limitations of comprehensiveness (e.g., antibody variation for identifying immune subsets). A new method for estimating immune cells according to the gene expression signature is worthwhile to overcome these issues. Nowadays, computational tools, including CIBERSORT and TIMER, have been developed for the high-throughput characterization of immune cells [27,28,29,30]. Petitprez et al., developed the device available for mouse studies by which accurate quantification of immune and stromal cell populations can be possible [31].

Wang et al., studied the immune microenvironment using a large-scaled sample pair obtained from 96 primary and recurrent GBM patients [32]. They collected gene expression profiles by transcriptome sequencing and made them available through a web portal (http://recur.bioinfo.cnio.es/ ) associated with GlioVis (http://gliovis.bioinfo.cnio.es/) [33]. Then, the tumor microenvironment was compared between primary and recurrent GBMs using CIBERSORT. The results showed a decreased monocyte proportion in rGBMs, suggesting relative depletion of circulation-derived monocytes. They further looked into the immune proportions in line with the GBM subtypes: Proneural (TCGA-PN), Neural (TCGA-N), Classical (TCGA-CL), and Mesenchymal (TCGA-MES) defined by The Cancer Genome Atlas (TCGA) [34]. In patients with MES-GBMs, which recurred from non-MES (PN or CL) ones (i.e., N.MES to MES transition), the high proportion of M2 macrophage was observed. Additionally, compared with primary MES-GBMs, recurrent ones showed an increase in non-polarized M0 macrophages and dendritic cells. The proportion of CD8+ T cells was significantly increased in hypermutated recurrent patients compared with the paired primary phase. Therefore, CIBERSORT is a remarkable application system to predict the immune signatures between different subtypes or progression stages of tumors. 

Recent scRNAseq analyses by Neftel et al., have newly identified the four cellular states of GBM cells, which recapitulate the gene expression modules of the neural-progenitor-like (NPC-like), oligodendrocyte-progenitor-like (OPC-like), astrocyte-like (AC-like), and mesenchymal-like (MES-like) (which is not anchored in neurodevelopment) [35,36]. Interestingly, they demonstrated that overexpression of CDK4 gene promoted the proliferation of mouse neural progenitor cells but not in astrocytes. On the other hand, overexpression of the EGFR gene promoted such a cancerous feature in astrocytes but not in neural progenitor cells. Thus, each state has a specific alteration of oncogenes, respectively. Some GBMs have multiple gene expression modules of these four cellular states, accounting for their plasticity and heterogeneity in a tumor, i.e., each of the tumors contains cells in at least two of the four cellular states and most tumors consist of all four. The most dominant state in a tumor is definitely linked to the previously established subtypes in TCGA; CL and MES subtypes correspond to AC-like and MES-like states, and the PN to OPC-like and NPC-like states, respectively [35]. Considering that the subtype transition may occur upon recurrence and the microenvironmental stress by therapeutic interventions deeply associates this transition [32], elucidation of the interplay between host immune cells and individual subtype of tumor cells and their dynamics along the treatments will be essential to unravel the precise mechanism of GBM recurrence [37]. 

### 3.2. Murine Models That Mimic GBM Recurrence

Recent omics analysis deepens our understanding of the immune microenvironment in the late phase of GBM progression and recurrence [38,39], but as the microenvironment may progressively change along with cancer recurrence [40], it is of great interest to what is happening at the early phase of recurrence and triggers cancer regrowth.

Liu et al., established one orthotopic murine recurrent model of intracranial tumor mass that had macroscopically been removed and undergone chemo/radiotherapy to maximize survival benefits [41]. This experimental mimic of the therapeutic process extended the median survival and revealed the involvement of the Akt/vimentin signaling in GBM recurrence. Interestingly, after surgical operation, astrocytes were highly positive for GFAP protein (i.e., in an activated state) and expressed PD-L1 around the tumor-infiltrating foci and invasive front, suggesting that host astrocytes play an essential role in immunosuppression of GBM recurrence after surgery [41]. On the other hand, Zhao et al., imitated recurrence using a genetically engineered murine GBM cell model with the HSV-TK suicide gene, in which the cell death program is activated after ganciclovir administration, allowing initial tumor expansion and partial regression in the deep area of the brain [42], and then revealed the increased invasiveness of rGBM cells and a higher ratio of monocyte-derived macrophages among the entire population of tumor-associated myeloid cells. Patient individuality, such as race, culture, and history of treatments impacts the features of the microenvironment, which may make it challenging to identify the key targets accurately. To overcome these issues, further development of sophisticated models that recapitulate GBM recurrence must be imperative. In addition, murine models must be indispensable to trace the precise dynamics of how GBM becomes resistant to various kinds of therapies, including molecular-target therapies [43].

## 4. Cancer Stem Cell (CSC) in Recurrent GBMs

The major impediment to the development of effective cancer therapies is the heterogeneity of tumor cells, especially genomic diversity caused by cancer evolution and the surrounding stromal components. The cancer stem cell (CSC) theory states that tumor growth is initiated from a small subpopulation of “rogue” cells located at the top of the cancer cell hierarchy, which differentiate into their progenies that constitute tumor mass. These rogue cells have often been reported as CSCs with stem cell-like properties and serve as the driving force for cancer initiation and evolution. In GBMs as well as many other types of cancers, CSCs have been considered a potential therapeutic target for eradication, consistent with the roles in recurrence due to their dominant contribution to therapy resistance [44].

The recent scRNAseq reproved the presence of glioma CSCs (GSCs) and the cellular hierarchy. In IDH-mutant oligodendrogliomas and astrocytomas, it is demonstrated that the proliferation ability is restricted mainly to neural progenitor cell (NPC)-like cells with a high stemness score. On the other hand, in pediatric gliomas carrying histone H3 lysine 27 to methionine mutant (H3K27M gliomas), the putative proliferating GSCs with high stemness scores are OPC-like states, suggesting that the different cells of origin drive the different types of gliomas. Notably, the proliferating signatures were observed in all four cellular states in GBMs and each state has a typical expression of well-known GSC markers. In other words, different GSC markers isolate GSCs from different cellular states. Based on these observations, Suva et al., propose that comprehensive analyses integrating all approaches from genetic to functional, including transcriptome, DNA methylome, and mitochondrial mutation analysis, could provide significant insight into the fundamental rules underlying GSC-driven tumor initiation [45,46].

In the context of GBM recurrence, the presence of residual tumor cells beyond the surgical margins and their resistance to postsurgical treatments is an urgent issue to be solved [47,48,49,50,51]. Increasing evidence has accumulated that GSCs isolated from invading edges have distinct properties with those from the core [52,53,54,55]. The microenvironment at the tumor margin is composed of astrocytes, microglia, oligodendrocytes, and stromal cells, speculating that GSC reconstructs the surrounding microenvironment involved in recurrence as the GSC microenvironment (i.e., GSC niche) plays an important role in cancer progression and the maintenance of its natures, although there is increasing evidence that surgical intervention as well as chemo/radiotherapies rearrange the microenvironment. 

**Figure 1:**
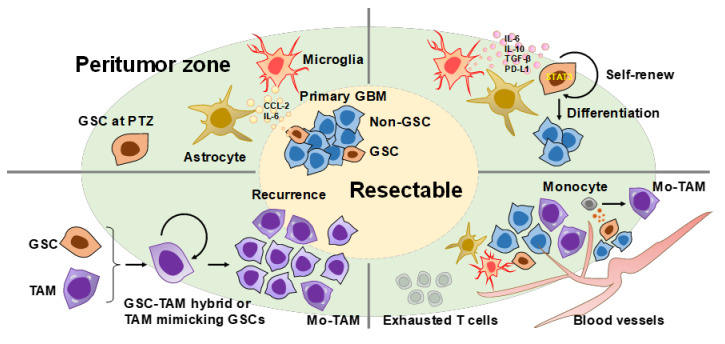
The putative footsteps of how GSCs complete recurrence. In primary GBM, GSCs release cues (e.g., CCL-2 and IL-6) to induce reactive astrocytes and microglia (top left). Surgical resection can remove the primary tumor at the resectable zone. However, GSCs remained at the peritumor zone (PTZ). GSCs at PTZ receive cues (e.g., IL-6, IL-10, TGF-β, and PD-L1) from reactive glial cells and start to regrow or self-renew to generate their differentiated progenies (top right). Once GSCs have grown to a certain extent, GSCs arrange their suitable microenvironment by recruiting monocytes derived from bone marrow and mediate their differentiation into tumor-associated macrophages (TAM). A variety of immune cells are infiltrated in the GBM tissue, but the anti-tumor immune compartments are mostly exhausted (bottom right). GSCs are further being progressive with the virtue of not well-proven mechanisms (e.g., TAM–GBM hybrids) to complete recurrence (bottom left).

Given that the marginal tumor region is adjacent to non-neoplastic cells such as astrocytes and microglia, GSCs at the peritumor zone (PTZ) may receive extracellular cues from them to self-renew and generate progenitors as the initial step of recurrence [56]. As intensively reviewed elsewhere [2,57,58,59], GBM releases various kinds of secreted factors to induce the transformation of astrocytes and microglia into reactive ones, which in turn release CCL2 and interleukin-6 (IL-6) toward GSCs (Figure 1, top left). For example, IL-6 produced by these glial cells supports the growth of GSCs via STAT3 activation (Figure 1, top right) [60,61]. These non-neoplastic cells provide a GSC-friendly immunosuppressive microenvironment via the expression of programmed death-ligand 1 (PD-L1) and the production of cytokines such as interleukin-10 (IL-10) and transforming growth factor-beta (TGF-β) [61]. On the other hand, once GSCs have grown to a certain extent, they may transdifferentiate into vascular components such as endothelium and pericytes and may stand by for organizing a biological network of host immune cells [62,63,64] by secreting chemokines, e.g., CCL2, and for maintaining the immunosuppressive microenvironment [65,66]. Especially, the recruited Mo-TAMs are activated by some factors from GSCs and strongly contribute to GBM progression (Figure 1, bottom right) [67,68,69]. 

## 5. Beyond the Interactions between GSCs and Surrounding Immune Cells 

Not only via paracrine factors (i.e., cytokines and growth factors) from TAMs but GBM cells themselves have been reported to further contribute to the malignant progression of GBM immune microenvironments by forming “TAM–GBM hybrids” [11,12,13]. Especially, Cao et al., found the presence of the hybrids (defined by GFAP+ and CD68+ double-positive cells) in clinical GBM samples, and they also demonstrated the formation of the hybrid by co-culturing the murine GL261 GBM cell line with bone marrow-derived macrophages. The hybrids were further found to be associated with high invasion-related genes and to possess high invasion capacity assessed by a Matrigel invasion assay. They observed CD11b+ RFP (red fluorescence protein and double-positive cells (TAM–GBM hybrids) in tumors formed by orthotopic transplantation of GL261 cells labeled with RFP. Notably, such hybrids were more present in the invasive region than in the core one. Although the molecular basis was entirely unknown, these findings are fascinating in that the hybrid formation of ovarian cancer cells with hematopoietic stem cells can confer even cancer stemness [70]. The apparent infiltration of hematopoietic stem/progenitor cells was recently identified in GBMs, and their functions are to promote the expression of the immune checkpoint PD-L1 and secretion of tumor-promoting cytokines [71]. 

The additional mechanism recently reported by Gangoso et al., is that GBM cells emulate molecular characteristics of myeloid cells as “myeloid mimicry” to overcome host immune attacks. They demonstrated that GBM cells epigenetically change their gene expression profiles upon exposure to interferon-γ (IFN-γ), a known important effector molecule of anti-tumor immunity [10]. Several researchers have reported a high proportion of T cells in rGBM [17,26,32]. These findings may give us a caution in therapy-mediated reactivation of cytotoxic lymphocytes that triggers the production of IFN-γ, granzymes, and perforin, because they possibly affect the immune evasion capabilities of rGBM cells by inducing myeloid mimicry deployment. On the whole, anti-cancer immunity is exhausted in pGBM and rGBM, but we must keep in mind that anti-tumor immune components may paradoxically support tumor cells in some types of GBMs. 

Although it has not yet been clarified whether the above-mentioned “hybrid” formation and “myeloid mimicry” could be driven by GSCs, we should take a hard look at such unique characteristics of cancer cells as potential targets for the prevention of recurrence and the eradication of GBMs (Figure 1, bottom left). 

## 6. Conclusions and Future Perspectives

Recent research advances in the immune microenvironment in recurrent GBM (rGBM) have been summarized in this review. We have also discussed how the microenvironments that support cancer regrowth are configured in rGBM by glioblastoma cancer stem cells (GSCs). The immune microenvironment in rGBM is characterized by Mo-TAM enrichment and immunosuppression by non-neoplastic cells such as reactive astrocytes and microglia. Although primary GBMs (pGBMs) are also described as immunosuppressive, the situation seems to be exacerbated in rGBMs by reactive non-neoplastic cells exposed to extracellular cues released from GSCs, which in turn stimulate GSCs residing in the peritumoral zone after surgical resection. This process might be a very initial step for GBM recurrence and, therefore, should be appropriately unraveled in murine models. 

In addition, combining the databases such as GlioVis (http://gliovis.bioinfo.cnio.es/) and RecuR (http://recur.bioinfo.cnio.es/) with biological assays is fascinating to complement the limited number of samples in rGBMs. IVY GAP (https://glioblastoma.alleninstitute.org/) also provides us with spatial information of where genes of interest are expressed in GBM tissue sections [72]. Additionally, computational tools for understanding immune components are now widely utilized by combining them with TCGA databases. These efforts will undoubtedly promote future GBM research, which will lead us to new effective treatments against molecular networks beyond molecular targets. Finally, there still remain some peculiar phenomena regarding the missing puzzle pieces that are presumably associated with GBM malignancy [10,11,12,13]. Further investigation in consideration of “TAM–GBM hybrids” and deploying “myeloid mimicry” will expand our understanding of unique immune microenvironments in rGBMs.
